# Repetitive transcranial magnetic stimulation on chronic tinnitus: a systematic review and meta-analysis

**DOI:** 10.1186/s12888-020-02947-9

**Published:** 2020-11-23

**Authors:** Zhengrong Liang, Haidi Yang, Gui Cheng, Lingfei Huang, Tao Zhang, Haiying Jia

**Affiliations:** 1 Department of Otolaryngology, The First Affifiliated Hospital of Jinan University, 601 Huangpu Avenue, Guangzhou, 510632 China; 2 Department of Otolaryngology, Sun Yat-sen Memorial Hospital, Sun Yat-sen University, Guangzhou, China; 3 Hearing and Speech Department, Xinhua College of Sun Yat-sen University, Guangzhou, China

**Keywords:** Repetitive transcranial magnetic stimulation, Chronic tinnitus, Randomized controlled trial, Systematic review and meta-analysis

## Abstract

**Background:**

Although the clinical efficacy and safety of repetitive transcranial magnetic stimulation (rTMS) in the treatment of chronic tinnitus have been frequently examined, the results remain contradictory. Therefore, we performed a systematic review and meta-analysed clinical trials examining the effects of rTMS to evaluate its clinical efficacy and safety.

**Methods:**

Studies of rTMS for chronic tinnitus were retrieved from PubMed, Embase, and Cochrane Library through April 2020. Review Manager 5.3 software was employed for data synthesis, and Stata 13.0 software was used for analyses of publication bias and sensitivity.

**Results:**

Twenty-nine randomized studies involving 1228 chronic tinnitus patients were included. Compared with sham-rTMS, rTMS exhibited significant improvements in the tinnitus handicap inventory (THI) scores at 1 week (mean difference [MD]: − 7.92, 95% confidence interval [CI]: − 14.18, − 1.66), 1 month (MD: -8.52, 95% CI: − 12.49, − 4.55), and 6 months (MD: -6.53, 95% CI: − 11.406, − 1.66) post intervention; there were significant mean changes in THI scores at 1 month (MD: -14.86, 95% CI: − 21.42, − 8.29) and 6 months (MD: -16.37, 95% CI: − 20.64, − 12.11) post intervention, and the tinnitus questionnaire (TQ) score at 1 week post intervention (MD: -8.54, 95% CI: − 15.56, − 1.52). Nonsignificant efficacy of rTMS was found regarding the THI score 2 weeks post intervention (MD: -1.51, 95% CI: − 13.42, − 10.40); the mean change in TQ scores 1 month post intervention (MD: -3.67, 95% CI: − 8.56, 1.22); TQ scores 1 (MD: -8.97, 95% CI: − 20.41, 2.48) and 6 months (MD: -7.02, 95% CI: − 18.18, 4.13) post intervention; and adverse events (odds ratios [OR]: 1.11, 95% CI: 0.51, 2.42). Egger’s and Begg’s tests indicated no publication bias (*P* = 0.925).

**Conclusion:**

This meta-analysis demonstrated that rTMS is effective for chronic tinnitus; however, its safety needs more validation. Restrained by the insufficient number of included studies and the small sample size, more large randomized double-blind multi-centre trials are needed for further verification.

## Background

Tinnitus is a common auditory symptom that brings severe psychological stress to patients and is associated with co-existing symptoms, such as hearing loss, dizziness, and concentration problems. Studies have estimated that the incidence of tinnitus in adults ranges from 10 to 19% [1, 2], and it is characterized by an experience of abnormal auditory perception in the head or ear in the absence of external acoustic or electrical stimulation. The 2019 European Multidisciplinary Tinnitus Guidelines defined it as chronic when patients have experienced related symptoms for more than 6 months [3]. Long-term tinnitus is not only annoying but often causes different degrees of mood disorders. Estimates have shown that in 1-3% of these patients, their quality of life had seriously deteriorated [1]. A study examining a neurophysiological model of tinnitus revealed abnormal electrical activities of neurons in the peripheral and central auditory pathways (including the cerebral cortex), resulting in effective auditory detection and insights into the processing of sound perception in the cortex or subcortical centre in tinnitus [4].

In recent years, there has been a growing annual prevalence of tinnitus, which might be related to the lack of a cure for most patients and a lack of effective standardized treatments. Several studies have ascertained supportive evidence that rTMS is effective in the treatment of chronic tinnitus [5, 6]. rTMS is a non-invasive technique that involves electromagnetic pulses passing through the skull into the brain that can reduce the excitability of relevant neurons and neurotransmitter systems in tinnitus [7].

Theoretically, hyperactive auditory neurons in the hearing centre can be adjusted through rTMS, thus reducing the occurrence of tinnitus and showing treatment efficacy. Although the clinical efficacy and safety of rTMS in chronic tinnitus have recently been reported, the results have been divergent and even contradictory. The efficacy of rTMS on chronic tinnitus was first systematically reviewed in 2011; the review included 5 randomized studies and concluded that rTMS was useful for tinnitus [8]. However, this review was limited due to the specificity of the population, a sample size (233 enrolled patients) that was quite small, and the inability to perform a quantitative analysis [8]. Several subsequent systematic reviews that evaluate rTMS have also reported similar problems [9, 10]. The most recent systematic review, which incorporated 15 studies, showed that rTMS treatment had a significant effect on tinnitus. In this review, similar issues emerged as there were only a few studies included in the quantitative analysis, which was insufficient for assessment of publication bias and sensitivity analysis, so the reliability of the conclusion was uncertain [11]. Beyond the evaluation of efficacy, none of the previous systematic evaluations or meta-analysis studies have quantitatively analysed the safety of rTMS [8–11].

In this study, we retrieved the published literature on rTMS as a treatment for chronic tinnitus and extracted highly relevant data to meta-analyse its efficacy and safety. This study provides a reference and encourages more clinical studies for the treatment of chronic tinnitus.

## Methods

### Search strategies

This study was executed in line with the guidelines of the Preferred Reporting Items for Systematic Reviews and Meta-Analyses (PRISMA) [12] and reported based on the guidelines developed by the Meta-Analysis of Observational Studies in Epidemiology group [13]. Because our analyses were performed based on previous studies, ethical approval and patient informed consent were not required. In the initial screening, two investigators (Z-RL and GC) independently conducted database searches in PubMed, Embase, and Cochrane Library to retrieve randomized controlled trials (RCTs) evaluating the efficacy and safety of rTMS for chronic tinnitus that were published from database inception to April 2020, without restrictions to languages or regions. Combined Medical Subject Headings (MeSH) and non-MeSH terms were searched as follows: transcranial magnetic stimulation, transcranial magnetic stimulations, magnetic stimulation AND transcranial, magnetic stimulations AND transcranial, stimulation AND transcranial magnetic, stimulations AND transcranial magnetic, transcranial magnetic stimulation AND single pulse, transcranial magnetic stimulation AND paired-pulse, transcranial magnetic stimulation AND repetitive, tinnitus, ringing-buzzing-tinnitus, ringing buzzing tinnitus, tinnitus AND tensor palatini induced, tensor palatini induced tinnitus, tinnitus AND tensor tympani induced, tensor tympani induced tinnitus, pulsatile tinnitus, tinnitus AND pulsatile, tinnitus AND spontaneous otoacoustic emission, tinnitus AND spontaneous otoacoustic emission, spontaneous otoacoustic emission tinnitus, spontaneous otoacoustic emission tinnitus, tinnitus AND clicking, clicking tinnitus, tinnitus AND Leudet, Leudet tinnitus, tinnitus AND Leudet’s, Leudet’s tinnitus, tinnitus AND Leudets, tinnitus AND noise-induced, induced tinnitus AND noise, noise-induced tinnitus, tinnitus AND objective, objective tinnitus, tinnitus AND subjective, subjective tinnitus, tinnitus of vascular origin, tinnitus of vascular origin, vascular origin tinnitus, tinnitus AND vascular origin. A third investigator not involved in the initial procedures was consulted in case of any discrepancies.

### Eligibility criteria

Two independent investigators (Z-RL and GC) analysed the initially selected articles to verify their relevance to the topic of rTMS as the treatment for chronic tinnitus. Studies were included if they (i) reported the clinical efficacy and safety of rTMS in chronic tinnitus, (ii) were RCTs, and (iii) recruited participants without limitations to regions, ages, or social status. Studies were excluded if they fulfilled the following criteria: non-randomized controlled studies, duplicate trials or overlapping data, animal experiments, conference abstracts, letters, and review articles. In case of any disagreement, the results were discussed and a decision made by the senior authors.

### Data extraction

Data were extracted from the eligible studies and independently categorized by two authors (Z-RL and GC) using a predefined data extraction form. All disagreements were resolved by discussion. The study design, baseline characteristics of the population (mean age, sample size, course of the disease, and country), interventions, scores for clinical efficacy, adverse events, and others were stratified into the rTMS and control groups using a standardized evidence table. All data were cross-checked to ensure accuracy. The procedures for study selection are shown in the PRISMA flow diagram.

### Methodological quality assessment

The methodological quality of the included studies was evaluated by two independent reviewers (Z-RL and GC) using Cochrane Handbook Version 5.3 from six dimensions: random sequence generation; allocation concealment; blinding of participants, personnel, and outcome assessors; incomplete outcome data reporting; selective reporting of outcomes; and other sources of bias.

### Statistical analysis

The meta-analysis and statistical analysis were performed using Cochrane Collaboration Review Manager software (RevMan version 5.3, Nordic Cochrane Center, Copenhagen, Denmark). We used the risk ratios (RRs) or odds ratios (ORs) for comparisons of dichotomous variables and the weighted mean difference (WMD) for comparisons of continuous variables. The *I*-square (*I*^2^) test was performed to assess the influence of heterogeneity on the output of the meta-analysis. *I*^2^ statistics of 0, 25, 50, and 75% corresponded to no, low, medium, and high heterogeneity, respectively. According to the Cochrane review guidelines, a random effects model was used when *I*^2^ ≥ 50% (high heterogeneity). Otherwise, a fixed effects model was used. A *P-*value of less than 0.05 was accepted as the threshold for statistical significance. The leave-one-out sensitivity analysis [14] was conducted by removing one study at a time to evaluate the quality and consistency of the results. Publication bias was visually assessed by funnel plots and Egger’s and Begg’s linear regression tests using Stata 13.0 software.

## Results

### Study selection process

**Fig. 1:**
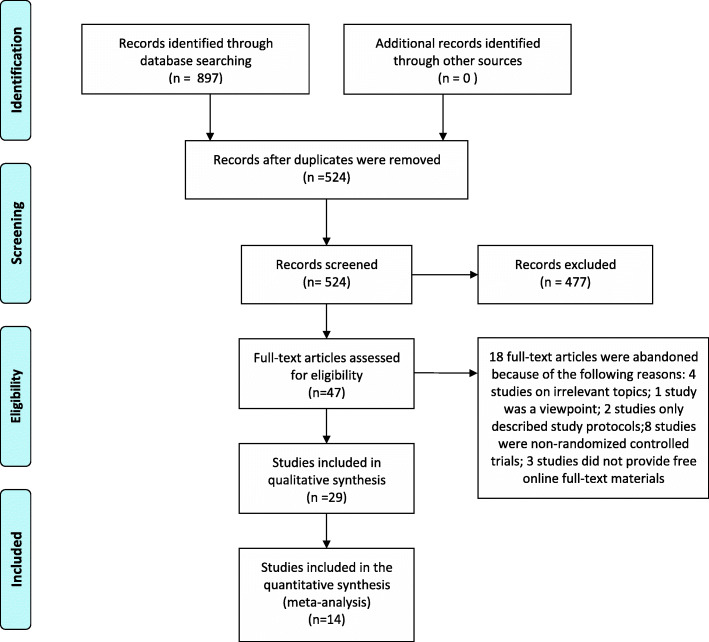
The flow diagram of the literature search and study selection

During our database search, 897 studies were initially retrieved, and 524 were selected after eliminating duplicates. Then, 477 studies without high relevance to our topic were discarded after reading titles and abstracts, and 47 studies were further evaluated by reading the full manuscripts. As a result, 18 full-text articles were abandoned for the following reasons: 4 described topics irrelevant to the efficacy and safety of rTMS on chronic tinnitus; 1 was a viewpoint; 2 were protocol designs; 8 were non-randomized controlled studies; and 3 did not provide free online full-text materials. Ultimately, 29 RCTs with 1228 patients were included in this systematic review and meta-analysis. The flow chart depicting the study selection process is shown in Fig. 1.

### Study characteristics and methodological quality

**Fig. 2:**
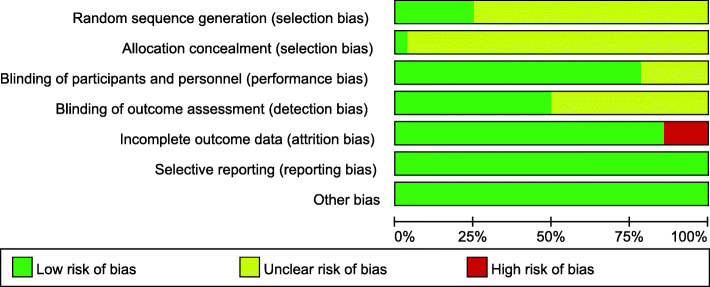
Risk of bias graph

**Fig. 3:**
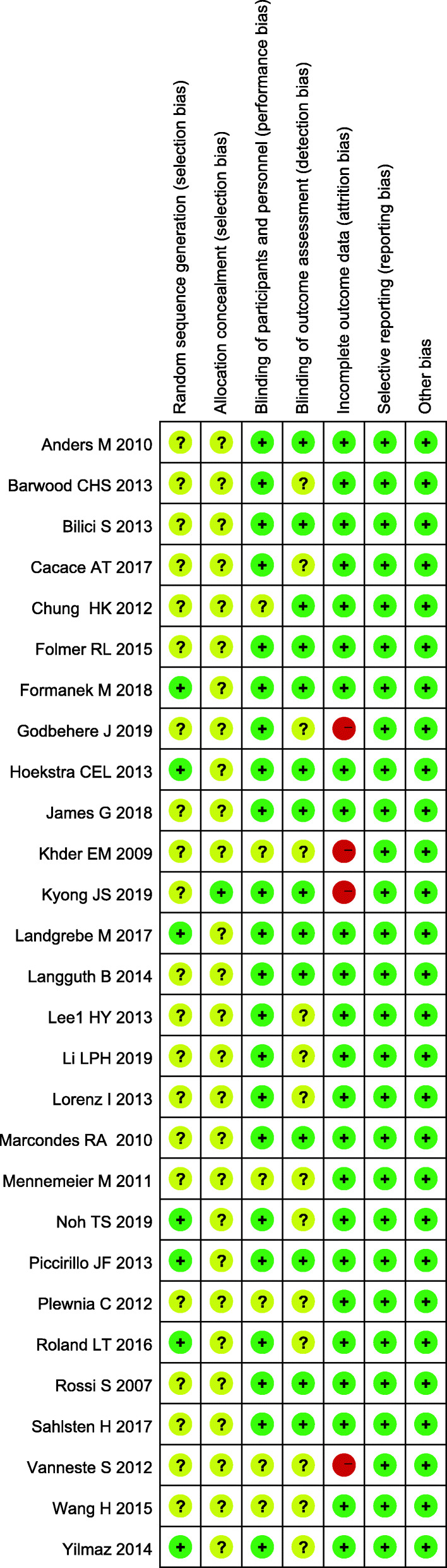
Risk of bias summary

The 29 eligible studies were randomized controlled studies published from 2004 to 2017. Six were conducted in the USA, 4 in Germany, 3 in China (including 1 in Taiwan), Turkey, and South Korea, 2 in the Czech Republic, and 1 in Italy, Egypt, Brazil, Australia, Netherlands, Finland, the UK, and Belgium. These clinical trials exhibited sample sizes that varied between 8 and 146 participants, a mean duration of tinnitus between 6 and 420 months, and a mean treatment course between 5 and 20 days. Of the 29 studies included (34 comparisons), 27 studies (32 comparisons) assessed the auditory cortex, 1 examined the motor cortex, and 1 did not target a specific cerebral area. Among the 32 comparisons in the 27 studies focusing on the auditory cortex, 19 comparison analyses showed the superiority of rTMS over sham-rTMS. Additionally, the 1 study focusing on the motor cortex confirmed the advantage of rTMS compared to sham-rTMS. In terms of the number of rTMS sessions, 11 studies (15 comparisons) reported a treatment time of 10 days, 12 (12 comparisons) of 5 days, 4 (4 comparisons) of 20 days, 1 (1 comparison) of 4 days, and 1 (2 comparisons) did not provide the stimulation duration. Regarding insights into different courses of rTMS treatment, 9 comparison analyses about a 5-day treatment showed that rTMS had better efficacy than sham-rTMS; however, the advantage of rTMS was nonsignificant after 20 days of treatment in all studies. Of the 29 studies (34 comparisons) included, 20 (23 comparisons) explored the left auditory cortex in patients with unilateral or bilateral tinnitus. In all eligible studies, 2 included only patients with bilateral tinnitus, 3 did not describe the tinnitus-affected side, and the remaining 24 included patients with either unilateral or bilateral tinnitus. Fifteen studies (18 comparisons) reported hearing loss in some or all of the included patients. The basic characteristics of the 29 studies are summarized in Table 1. The methodological quality graphs (Figs. 2 and 3) presented each item for each included study, and each item was shown as percentages across all trials according to our established quality evaluation standard.

**Table 1:** Characteristics of the included studies

Included trials	Country	Interventions		Studydesign	Gender (male/female)		Age (years)		Duration of tinnitus (month)		Stimulation site	Treatment course (days)	Follow-up length	Conclusion by author
		T	C		T	C	T	C	T	C				
Landgrebe M 2017 [15]	Germany	1-Hz rTMS(2000 stimuli, 110% motor threshold)	sham rTMS	Sham-controlled, randomized multi-centre trial	54/17	51/24	48.1 ± 12.5	49.9 ± 13.2	6.2 ± 5.3	8.1 ± 8.4	Left temporal cortex	10 d	6 months	Nonsignificant
Formanek M 2018 [5]	Czech Republic	1-Hz rTMS(1000 stimuli, 110% motor threshold, the DLPFC on the left side and primary AC on both sides); 25-Hz rTMS (300 stimuli, 80% motor threshold, DLPFC)	sham rTMS	Randomized double-blind controlled trial	13/7	10/2	47.9 ± 14.31	51.8 ± 10.34	53.4 ± 61.89	76.8 ± 76.85	DLPFC on the left side and primary AC on both sides	5 d	6 months	Nonsignificant
Chung HK 2012 [16]	China	5-Hz rTMS(900 stimuli, 80% motor threshold)	sham rTMS	Parallel randomized control study	11/1	11/1	53.83 ± 18.4	51.90 ± 15.5	6-240	6-240	Left temporoparietal region	10 d	1 month	Significant
Yilmaz 2014 [17]	Turkey	1-Hz rTMS(1800 stimuli, motor threshold: unclear)	sham rTMS	Randomized controlled trial	27/33	27/33	49.8 ± 8.03 (36-66)	49.8 ± 8.03 (36-66)	> 6	> 6	Unclear	10 d	1 month	Significant
Rossi S 2007 [18]	Italy	1-Hz rTMS(1200 stimuli, 120% motor threshold)	sham rTMS	Randomized, double blind, crossover, placebo-controlled trial	7/1	4/2	52.63 (35-72)	52.33 (37-62)	12-300	12-300	Left temporoparietal region	5 d	6 weeks	Significant
langguth B 2014 (1) [19]	Germany	1-Hz rTMS(2000 stimuli, 110% motor threshold)	sham rTMS	Randomized, double-blind, parallel-group, controlled clinical trial	35/13	31/14	44.9 ± 11.5	50.3 ± 12.9	68.0 ± 97.0	74.4 ± 74.2	PET-based neuronavigation	10 d	11 weeks	Nonsignificant
langguth B 2014 (2) [19]	Germany	1-Hz rTMS(2000 stimuli, 110% motor threshold)	sham rTMS	Randomized, double-blind, parallel-group, controlled clinical trial	32/16	31/14	50.4 ± 12.5	50.3 ± 12.9	78.3 ± 64.9	78.3 ± 64.9	Left AC	10 d	11 weeks	Nonsignificant
Bilici S 2015 (1) [20]	Turkey	1-Hz rTMS (900 stimuli, 110% motor threshold)	sham rTMS	Randomized, double-blind, placebo-controlled study	33/42	33/42	40 ± 13.2 (20-62)	40 ± 13.2 (20-62)	> 12	> 12	Left temporoparietal region	10 d	6 months	Significant
Bilici S 2015 (2) [20]	Turkey	10-Hz rTMS(600 stimuli, 110% motor threshold)	sham rTMS	Randomized, double-blind, placebo-controlled study	33/42	33/42	40 ± 13.2 (20-62)	40 ± 13.2 (20-62)	> 12	> 12	Left temporoparietal region	10 d	6 months	Significant
Khedr EM 2009 (1) [21]	Egypt	1-Hz rTMS (1500 stimuli, 100% motor threshold)	sham rTMS	Randomized controlled trial	Unclear	Unclear	Unclear	Unclear	Unclear	Unclear	Left temporoparietal region	10 d	12 months	Nonsignificant
Khedr EM 2009 (2) [21]	Egypt	10-Hz rTMS (1500 stimuli, 100% motor threshold)	sham rTMS	Randomized controlled trial	Unclear	Unclear	Unclear	Unclear	Unclear	Unclear	Left temporoparietal region	10 d	12 months	Significant
Khedr EM 2009 (3) [21]	Egypt	25-Hz rTMS (1500 stimuli, 100% motor threshold)	sham rTMS	Randomized controlled trial	Unclear	Unclear	Unclear	Unclear	Unclear	Unclear	Left temporoparietal region	10 d	12 months	Significant
Marcondes RA 2010 [22]	Brazil	1-Hz rTMS (1020 stimuli, 110% motor threshold)	sham rTMS	Randomized, double-blind, parallel design, study	Unclear	Unclear	> 18	> 18	> 6	> 6	Left temporoparietal region	5 d	6 months	Significant
Folmer RL 2015 [23]	The USA	1-Hz rTMS (2000 stimuli, 110% or lower motor threshold)	sham rTMS	Randomized, participant and clinician or observer-blinded, placebo-controlled clinical trial	25/7	26/6	58.3 ± 9.5	62.8 ± 8.3	> 12	> 12	Left or right AC	10 d	6 months	Significant
Li LPH 2019 [24]	Taiwan, China	1-Hz rTMS (1800 stimuli, 110% or lower motor threshold)	sham rTMS	Randomized controlled trial	7/5	7/5	57 ± 10.1	54 ± 7.5	> 6	> 6	Left primary AC	5 d	1 month	Significant
Noh TS 2019 [25]	South Korea	1-Hz rTMS (2000 pulses over the AC and 1000 pulses over the DLPFC, 110% or lower motor threshold)	sham rTMS	Double-blind, randomized controlled trial	14/3	7/6	51.9 ± 12.4	55.8 ± 6.9	76.1 ± 129.3	70.1 ± 70.4	Left primary AC and left DLPFC	4 d	8 weeks	Significant
Anders M 2010 [26]	Czech Republic	1-Hz rTMS (1500 stimuli, 110% or lower motor threshold)	sham rTMS	Randomized, placebo controlled study	12/10	17/3	48.09	50.1	106.8 ± 81.6	88.4 ± 67.5	Left primary AC	10 d	6 months	Significant
Hoekstra CEL 2013 [27]	The Netherlands	1-Hz rTMS (2000 stimuli, 110% motor threshold)	sham rTMS	Randomized, double-blind placebo-controlled clinical trial	26/0	15/9	50 ± 12	50 ± 12	58 (8-240)	38 (12-420)	Unilateral AC	5 d	6 months	Nonsignificant
Sahlsten H 2017 [28]	Finland	1-Hz rTMS (4000 stimuli, 100% motor threshold)	sham rTMS	Randomized, placebo-controlled study	13/6	14/6	48.9 ± 13.1	51.5 ± 10.7	> 6	> 6	Left superior temporal gyrus	10 d	6 months	Nonsignificant
Wang H 2016 [29]	China	1-Hz rTMS (1000 stimuli, 110% motor threshold)	sham rTMS	Randomized controlled trial	6/8	3/7	62.1 ± 9.81	56.4 ± 11.8	6-72	6-72	Left temporoparietal region	10 d	Unclear	Significant
Cacace AT 2017 [30]	USA	1-Hz rTMS (1200 stimuli, 110% motor threshold)	sham rTMS	Randomized single-blinded sham-controlled crossover study	30/0	30/0	54.2 ± 14.2	54.2 ± 14.2	Unclear	Unclear	Left temporal cortex	5 d	Unclear	Significant
Piccirillo JF 2013 [31]	USA	1-Hz rTMS (1650 stimuli, 110% motor threshold)	sham rTMS	Crossover, double-blind, randomized controlled trial	9/5	9/5	Median 42 (22-59)	Median 42 (22-59)	6-360	6-360	Left temporoparietal area	20 d	> 4 weeks	Nonsignificant
James G 2018 [32]	USA	1-Hz or 10-Hz rTMS (1800 stimuli, 110% motor threshold)	sham rTMS	Double-blind, randomized clinical trial with participant crossover	9/3	9/3	49.2 ± 15.3	49.2 ± 15.3	> 6	> 6	Posterior superior temporal gyrus	5 d	Unclear	Significant
Kyong JS 2019 (1) [33]	South Korea	1-Hz rTMS (stimuli: unclear, motor threshold: unclear)	sham rTMS	Randomized controlled trial	4/4	6/2	56 ± 4.9	50.9 ± 7.1	> 6	> 6	Auditory temporal cortex	Unclear	Unclear	Nonsignificant
Kyong JS 2019 (2) [33]	South Korea	1-Hz rTMS (stimuli: unclear, motor threshold: unclear)	sham rTMS	Randomized controlled trial	6/2	6/2	50.9 ± 7.1	50.9 ± 7.1	> 6	> 6	Auditory temporal and the frontal regions	Unclear	Unclear	Significant
Roland LT 2016 [34]	USA	1-Hz rTMS (stimuli: unclear, 110% motor threshold)	sham rTMS	Randomized, double-blind, controlled clinical trial	11/5	10/4	Median: 50	Median: 53	> 6	> 6	Left temporoparietal junction	10 d or 20 d	4 weeks	Nonsignificant
Barwood CHS 2013 [35]	Australia	1-Hz rTMS (2000 stimuli, 110% motor threshold)	sham rTMS	Single-blind, randomized controlled trial	2/2	2/2	29-58		> 12	> 12	Left primary AC	10 d	3 months	Significant
Godbehere J 2019 [36]	UK	5-Hz rTMS (1200 stimuli, 80% motor threshold)	sham rTMS	Two-arm, single-blind, randomized controlled trial	Unclear	Unclear	Unclear	Unclear	Unclear	Unclear	Temporal-parietal region of the scalp, overlying the AC	5 d	4 weeks	Nonsignificant
Mennemeier M 2011 [37]	USA	1-Hz rTMS (1800 stimuli, 110% motor threshold)	sham rTMS	Randomized, sham-controlled crossover	Unclear	Unclear	28-75	28-75	> 6	> 6	Temporal cortex	5 d	Unclear	Significant
Lee HY 2013 [38]	South Korea	1-Hz rTMS (1200 stimuli, 100% motor threshold)	sham rTMS	Randomized controlled trial	8/7	8/7	53	53	Mean: 48	Mean: 48	The motor cortex	5 d	Unclear	Significant
Lorenz I 2013 [39]	Germany	1-Hz rTMS (1000 stimuli, 110% motor threshold)	sham rTMS	Randomized, single-blind, sham-controlled trial	7/3	7/3	49.8	49.8	Mean: 21.6	Mean: 21.6	Left AC	5 d	Unclear	Significant
Vanneste S 2012 [40]	Belgium	1-Hz or 10-Hz –rTMS (900 stimuli, 120% motor threshold)	sham rTMS	Randomized controlled trial	Unclear	Unclear	50.05 ± 11.77	50.05 ± 11.77	> 12	> 12	Left ventrolateral prefrontal cortex	5 d	12 months	Significant (for 10 Hz)
Plewnia C 2012(1) [41]	Germany	5-Hz-rTMS (2400 stimuli, 80% motor threshold)	sham rTMS	Randomized controlled trial	10/6	8/8	46.4 ± 13.0	45.6 ± 10.3	27 ± 14	22 ± 14	Bilateral secondary AC	20 d	12 weeks	Nonsignificant
Plewnia C 2012(2) [41]	Germany	5-Hz-rTMS (2400 stimuli, 80% motor threshold)	sham rTMS	Randomized controlled trial	7/9	8/8	55.8 ± 9.7	45.6 ± 10.3	28 ± 13	22 ± 14	Temporoparietal association cortex	20 d	12 weeks	Nonsignificant

*rTMS* repetitive transcranial magnetic stimulation, *AC* auditory cortex, *DLPFC* dorsolateral prefrontal cortex

### The clinical efficacy and safety of rTMS in the treatment of chronic tinnitus

#### THI scores 1 week post intervention

**Fig. 4:**
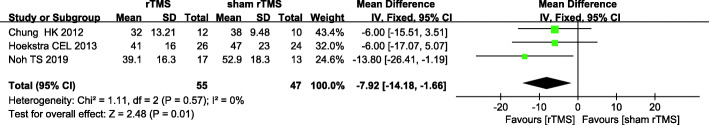
Comparisons of 1-week post-intervention THI scores between rTMS versus sham-rTMS groups

Of the 29 included studies, 3 reported [16, 25, 27] 1-week post-intervention THI scores. Because of nonsignificant heterogeneity (*I*^2^ = 0%, *P* = 0.57) among the studies, a fixed effects model was utilized. The outcome manifested a statistically significant difference between the rTMS and sham-rTMS groups (MD: -7.92, 95% CI: − 14.18, − 1.66; *P* = 0.01) (Fig. 4).

#### THI scores 2 weeks post intervention

Three studies [15, 25, 26] containing statistics for 1-week post-intervention THI scores were available for the analysis using a random effects model, with significant heterogeneity among the studies (*I*^2^ = 72%, *P* = 0.03). The results exhibited no statistically significant differences in the 2-week post-intervention THI scores between the two groups (MD:-1.51, 95% CI: − 13.42, 10.40; *P* = 0.80).

#### THI scores 1 month post intervention

**Fig. 5:**
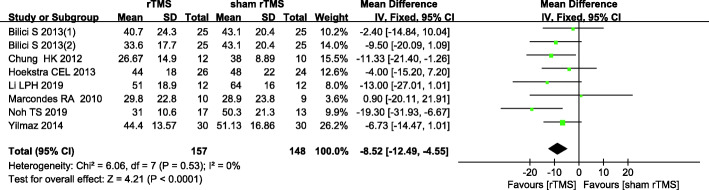
Comparisons of 1-month post-intervention THI scores between rTMS versus sham-rTMS groups

Seven studies [16, 17, 20, 22, 24, 25, 27] assessing 1-month post-intervention THI scores were included in the meta-analysis. There was no statistically significant heterogeneity among the studies (*I*^2^ = 0%, *P* = 0.53), so a fixed effects model was utilized. The results showed a significant difference in 1-month post-intervention THI scores between the two groups (MD: -8.52, 95% CI: − 12.49, − 4.55; *P* < 0.0001) (Fig. 5).

#### THI scores 6 months post intervention

**Fig. 6:**
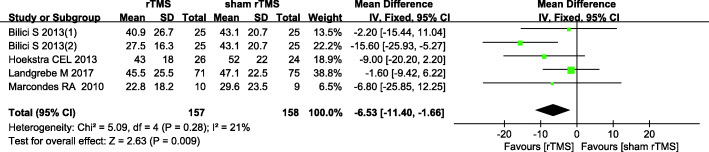
Comparisons of 6-month post-intervention THI scores between rTMS versus sham-rTMS groups

Four studies [15, 20, 22, 27] estimating 6-month post-intervention THI scores were available for the meta-analysis using a fixed effects model, with no statistically significant heterogeneity among the studies (*I*^2^ = 21%, *P* = 0.28). The results showed a significant difference in 6-month post-intervention THI scores between the two groups (MD: -6.53, 95% CI: − 11.40, − 1.66; *P* = 0.009) (Fig. 6).

#### Mean change in THI scores 1 month post intervention

Three studies [20, 22, 24] evaluating the mean change in THI scores from baseline to 1 month post intervention were meta-analysed using a random effects model, with significant heterogeneity among the studies (*I*^2^ = 56%, *P* = 0.08). The results exhibited a statistically significant difference in the mean change in THI scores at 1 month post intervention between the two groups (MD: -14.86, 95% CI: − 21.42, − 8.29; *P* < 0.00001).

#### Mean change in THI scores 6 months post intervention

Two studies [20, 22] evaluating the mean difference in THI scores from baseline to 6 months post intervention were meta-analysed using a fixed effects model, with no statistically significant heterogeneity among the studies (*I*^2^ = 0%, *P* = 0.87). The results showed that there was a significant difference in the mean change in THI scores 6 months post intervention between the two groups (MD: -16.37, 95% CI: − 20.64, − 12.11; *P* < 0.00001).

#### Other indicators for outcome evaluation

The following studies were meta-analysed for the outcome of patients: 2 [16, 27] appraising TQ scores 1 week post intervention; 2 [16, 27] with TQ scores 1 month post intervention; 2 [15, 27] with TQ scores 6 months post intervention; 3 [16, 19] (1 [19] containing two RCTs) with mean changes in TQ scores 1 week post intervention; 2 [17, 27] with VAS scores 1 month post intervention; and 2 [16, 17] with tinnitus loudness 1 month post intervention. There was a statistically significant difference in TQ scores 1 week post intervention between the rTMS and sham-rTMS groups (*P = 0.02*). Nonsignificant differences in other outcomes were found between the two groups (MD: -6.53, 95% CI: − 11.40, − 1.66; *P* = 0.009) (Table 2).

**Table 2:** Meta-analysis results of other indicators for outcome evaluation

Outcomes	Included studies (n)	Enrolled patients (T/C, n)	Heterogeneity	MD (95% CI)	*P*
TQ score 1 week post intervention	2	38/34	*P* = 0.55, I^2^ = 0%	-8.54 (−15.56, −1.52)	0.02
TQ score 1 month post intervention	2	38/34	*P* = 0.15, I^2^ = 53%	-8.97 (−20.41, 2.48)	0.12
TQ score 6 months post intervention	2	97/99	*P* = 0.03, I^2^ = 79%	-7.02 (−18.18, 4.13)	0.22
Mean change in TQ scores 1 week post intervention	3	108/100	*P* = 0.04, I^2^ = 69%	−3.67 (−8.56, 1.22)	0.14
VAS score 1 month post intervention	2	56/54	*P* = 0.07, I^2^ = 69%	−0.64 (−1.77, 0.48)	0.26
Tinnitus loudness 1 month post intervention	2	42/40	*P* = 0.71, I^2^ = 0%	−1.13 (−7.13, 4.87)	0.71

*TQ* tinnitus questionnaire, *VAS* visual analogue scale, *CI* confidence interval

#### Adverse events

**Fig. 7:**
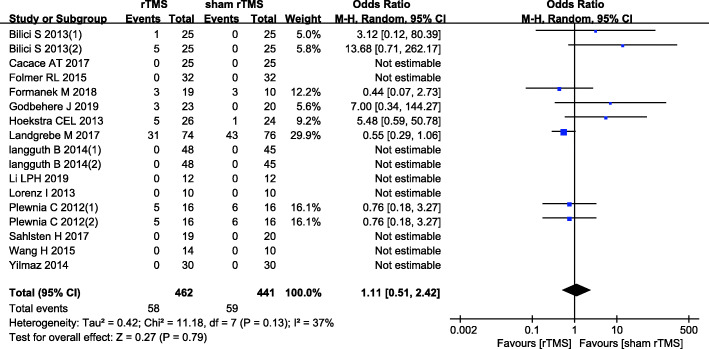
Comparisons of adverse events after treatment with rTMS versus sham-rTMS

Fifteen studies [5, 15, 17, 19, 20, 23, 27–31, 36, 39] reporting adverse events after rTMS sessions were meta-analysed using a fixed effects model, with nonsignificant heterogeneity among the studies (*I*^2^ = 37%, *P* = 0.13). The results showed a nonsignificant difference in the incidence of adverse events between the rTMS and sham-rTMS groups (12.55% vs. 13.38%; OR: 1.11, 95% CI: 0.51-2.42; *P* = 0.79) (Fig. 7). Among these adverse events, 21 patients reported headache; 7, worsening of tinnitus; and 5, sleep disturbances. Facial muscle discomfort, back pain, muscle hardening, and ENT symptoms (e.g., rhinitis, otitis media) were each reported in 3 patients; neck and shoulder stiffness and jaw spasms were each reported in 2 patients; increased sensitivity to noise, painful sensation in the affected ear, and anxiety and panic attacks were each reported in 1 patient. Nine patients reported other events.

#### Sensitivity analyses

**Fig. 8:**
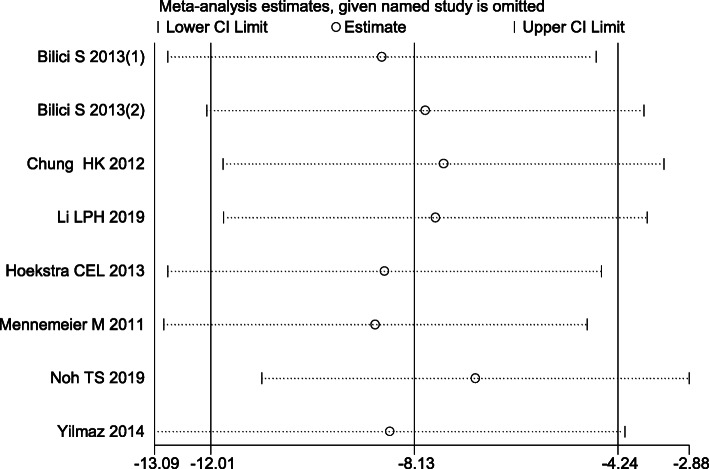
Sensitivity analysis for the stability of the results in the included studies

Sensitivity analyses were performed for the selected studies to identify outliers that affected the overall results. There was a nonsignificant difference in the stability of the results (Fig. 8), which validated the rationality and reliability of our meta-analysis.

#### Evaluation of publication bias

**Fig. 9:**
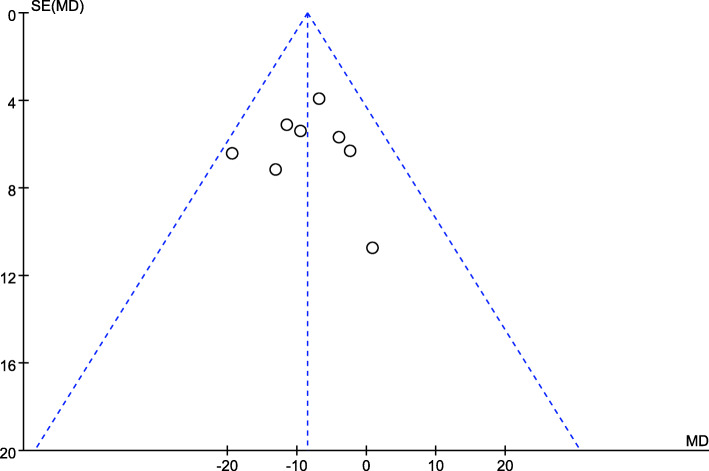
Funnel plot of the 1-month post-intervention THI scores

Visual inspection of funnel plots was adopted in this evaluation (Fig. 9). Egger᾽s and Begg᾽s analyses [16, 17, 20, 22, 24, 25, 27] showed no publication bias in our meta-analysis (*P* = 0.925).

## Discussion

In this study, we reported the results of a systematic review and meta-analysis of 29 selected RCTs showing that rTMS can effectively ameliorate chronic tinnitus. To ensure reliable conclusions, we retrieved, reviewed, and summarized previously published studies on rTMS for the treatment of chronic tinnitus, which had high quality and showed good compliance in patients, to answer various clinical questions about the efficacy and safety of this treatment. Overall, our results suggested that rTMS is effective for the treatment of chronic tinnitus. Subgroup analyses showed that rTMS started to exert its efficacy at 1 week and continued to be effective 6 months after treatment. In addition, rTMS is a safe option, as serious adverse events were evenly distributed between participants randomly assigned to the rTMS and sham rTMS groups. Of all included studies, 93.10% stimulated the auditory cortex as a predominant stimulation site, wherein 77.78% stimulated the left auditory cortex, regardless of which ear was affected. There is strong evidence that the left primary auditory cortex is a potential target for the 1-Hz-rTMS treatment of tinnitus in pilot studies [42]. This explains why most studies have chosen the left auditory cortex as a stimulus target, although a few studies did stimulate the contralateral side for unilateral chronic tinnitus. More than half of the studies reported hearing loss in some or all of the included patients, but there was no further analysis of whether hearing loss was related to tinnitus. The duration of rTMS treatment varied among studies, and the 5- (41.38%) and 10-day (37.93%) periods were the more common options. The 1-Hz stimulation was most frequently used (93.10%).

There is a high level of heterogeneity in tinnitus in the population. Although many clinical studies involving various treatments for tinnitus have been conducted, there is a lack of widespread agreement on its efficacy in reducing tinnitus loudness and the impacts of tinnitus, which might be attributed to the low level of evidence that cannot be used to verify the effects [43]. This poses a huge challenge to ear, nose, and throat (ENT) doctors. Landgrebe et al. found that daily low-frequency rTMS exhibited a cumulative effect on chronic tinnitus that not only caused synaptic inhibition and changes in synaptic plasticity in the auditory cortex but also improved haemodynamics in the auditory cortex [15]. Our findings regarding the efficacy of rTMS in chronic tinnitus are consistent with previous systematic evaluations and meta-analyses [8–11]. Moreover, our meta-analysis showed a nonsignificant difference in safety between rTMS and sham-rTMS.

Compared with previous meta-analyses associated with rTMS and tinnitus, our study has the following advantages. First, we performed a series of assessments for the included studies to ensure the high reliability of our conclusions, including publication of the protocol, detailed and predefined sensitivity, comprehensive assessments for risks of systematic and random errors, and assessments for the quality of evidence. Second, most of the studies were single- or double-blind studies with a relatively high level of evidence, which increased the reliability of the results. Third, the rationality and reliability of our meta-analysis have been prudently and significantly improved in that the overall comprehensive estimation has been performed based on a large sample size. Additionally, sufficient sensitivity analyses and the assessment of publication bias were carried out to ensure the stability of this meta-analysis. Fourth, we conducted a quantitative analysis of the safety of rTMS for the treatment of chronic tinnitus.

In addition, some limitations of our study must be acknowledged. First, despite the inclusion of recent large randomized trials, the limited number of enrolled subjects in our meta-analysis limits more accurate analyses, and some results were nonsignificant, which might be attributed to the nature of the population receiving rTMS. Second, this study only analysed English-language references, which leads to lost data from those in other languages. Third, although Egger᾽s and Begg᾽s analyses showed no publication bias in our meta-analysis, because of the limited number of studies included in this analysis, the possibility of false negatives cannot be excluded.

## Conclusion

In summary, our systematic review and meta-analysis confirms the efficacy of rTMS and shows satisfactory safety in patients with chronic tinnitus. However, its safety needs to be verified in large-sample studies. Restrained by the insufficient number of eligible studies and the nature of the target population, more proposals to encourage large-sample, multi-centre, randomized double-blind trials are needed for further verification.

## Data Availability

All data generated or analysed during this study are included in this published article and the original studies’publications.

## Abbreviations

rTMS: Repeated transcranial magnetic stimulation
CI: Condidence interval
THI: Tinnitus handicap inventory
TQ: Tinnitus questionnaire
PRISMA: Preferred Reporting Items for Systematic Reviews and Meta-Analyses
MeSH: Medical Subject Headings
AC: Auditory cortex
DLPFC: Dorsolateral prefrontal cortex
VAS: Visual analogue scale
CI: Confidence interval

## References

[1] Axelsson A, Ringdahl A (1989). Tinnitus-a study of its prevalence and characteristics. Br J Audiol.

[2] Gilles A, De Ridder D, Van Hal G (2012). Prevalence of leisure noise-induced tinnitus and the attitude toward noise in university students. Otol Neurotol.

[3] Cima R, Mazurek B, Haider H (2019). A multidisciplinary European guideline for tinnitus: diagnostics, assessment, and treatment. HNO.

[4] Jastreboff PJ (1990). Phantom auditory perception (tinnitus): mechanisms of generation and perception. Neurosci Res.

[5] Formánek M, Migaľová P, Krulová P (2018). Combined transcranial magnetic stimulation in the treatment of chronic tinnitus. Ann Clin Transl Neurol.

[6] Sahlsten H, Holm A, Rauhala E (2019). Neuronavigated versus non-navigated repetitive Transcranial magnetic stimulation for chronic tinnitus: a randomized study. Trends Hear.

[7] May A, Hajak G, Ganssbauer S (2007). Structural brain alterations following 5 days of intervention: dynamic aspects of neuroplasticity. Cereb Cortex.

[8] Meng Z, Liu S, Zheng Y (2011). Repetitive transcranial magnetic stimulation for tinnitus. Cochrane Libr.

[9] Peng Z, Chen XQ, Gong SS (2012). Effectiveness of repetitive transcranial magnetic stimulation for chronic tinnitus: a systematic review. Otolaryngol Head Neck Surg.

[10] Theodoroff SM, Folmer RL (2013). Repetitive transcranial magnetic stimulation as a treatment for chronic tinnitus: a critical review. Otol Neurotol..

[11] Soleimani R, Jalali MM, Hasandokht T (2016). Therapeutic impact of repetitive transcranial magnetic stimulation (rTMS) on tinnitus: a systematic review and meta-analysis. Eur Arch Otorhinolaryngol.

[12] Moher D, Liberati A, Tetzlaff J (2009). Preferred reporting items for systematic reviews and meta-analyses: the PRISMA statemen. BMJ.

[13] Stroup DF, Berlin JA, Morton SC (2000). Meta-analysis of observational studies in epidemiology: a proposal for reporting. JAMA.

[14] Fox MW, Piepgras DG, Bartleson JD (1995). Anterolateral decompression of the atlantoaxial vertebral artery for symptomatic positional occlusion of the vertebral artery: case report. J Neurosurg.

[15] Landgrebe M, Hajak G, Wolf S (2017). 1-Hz rTMS in the treatment of tinnitus: a sham-controlled, randomized multicenter trial. Brain Stimul.

[16] Chung HK, Tsai CH, Lin YC (2012). Effectiveness of Theta-burst repetitive Transcranial magnetic stimulation for treating chronic tinnitus. Audiol Neurotol.

[17] Yilmaz M, Yener MH, Turgut NF (2014). Effectiveness of transcranial magnetic stimulation application in treatment of tinnitus. J Craniofac Surg.

[18] Rossi S, De CA, Ulivelli M (2007). Effects of repetitive transcranial magnetic stimulation on chronic tinnitus: a randomised, crossover, double blind, placebo controlled study. J Neurol Neurosurg Psychiatry.

[19] Langguth B, Landgrebe M, Frank E (2014). Efficacy of different protocols of transcranial magnetic stimulation for the treatment of tinnitus: Pooled analysis of two randomized controlled studies. World J Biol Psychiatry.

[20] Bilici S, Yigit O, Taskin U (2015). Medium-term results of combined treatment with transcranial magnetic stimulation and antidepressant drug for chronic tinnitus. Eur Arch Otorhinolaryngol.

[21] Khedr EM, Rothwell JC, El-Atar A (2009). One-year follow up of patients with chronic tinnitus treated with left temporoparietal rTMS. Eur J Neurol.

[22] Marcondes RA, Sanchez TG, Kii MA (2010). Repetitive transcranial magnetic stimulation improve tinnitus in normal hearing patients: a double-blind controlled, clinical and neuroimaging outcome study. Eur J Neurol.

[23] Folmer RL, Theodoroff SM, Casiana L (2015). Repetitive Transcranial magnetic stimulation treatment for chronic tinnitus: a randomized clinical trial. JAMA Otolaryngol Head Neck Surg.

[24] Li LP, Shiao AS, Li CT (2019). Steady-state auditory evoked fields reflect long-term effects of repetitive transcranial magnetic stimulation in tinnitus. Clin Neurophysiol.

[25] Noh TS, Kyong JS, Park MK (2019). Treatment outcome of auditory and frontal dual-site rTMS in tinnitus patients and changes in Magnetoencephalographic functional connectivity after rTMS: double-blind randomized controlled trial. Audiol Neurootol.

[26] Anders M, Dvorakova J, Rathova L (2010). Efficacy of repetitive transcranial magnetic stimulation for the treatment of refractory chronic tinnitus: a randomized, placebo controlled study. Neuro Endocrinol Lett.

[27] Hoekstra CEL, Versnel H, Neggers SFW (2013). Bilateral low-frequency repetitive transcranial magnetic stimulation of the auditory cortex in tinnitus patients is not effective: a randomised controlled trial. Audiol Neurootol.

[28] Sahlsten H, Virtanen J, Joutsa J (2017). Electric field-navigated transcranial magnetic stimulation for chronic tinnitus: a randomized, placebo-controlled study. Int J Audiol.

[29] Wang H, Li B, Wu HM (2016). Combination of gaps in noise detection and visual analog scale for measuring tinnitus components in patients treated with repetitive transcranial magnetic stimulation. Auris Nasus Larynx.

[30] Cacace AT, Hu JN, Romero S (2017). Glutamate is down-regulated and tinnitus loudness-levels decreased following rTMS over auditory cortex of the left hemisphere: a prospective randomized single-blinded sham-controlled cross-over study. Hear Res.

[31] Piccirillo JF, Kallogjeri D, Nicklaus J (2013). Low-frequency repetitive transcranial magnetic stimulation to the temporoparietal junction for tinnitus: four-week stimulation trial. JAMA Otolaryngol Head Neck Surg..

[32] James GA, Thostenson JD, Brown G (2017). Neural activity during attentional conflict predicts reduction in tinnitus perception following rTMS. Brain Stimul..

[33] Kyong JS, Noh TS, Park MK (2019). Phantom Perception of Sound and the Abnormal Cortical Inhibition System: An Electroencephalography (EEG) Study. Ann Otol Rhinol Laryngol.

[34] Roland LT, Peelle JE, Kallogjeri D (2016). The effect of noninvasive brain stimulation on neural connectivity in tinnitus: a randomized trial. Laryngoscope.

[35] Barwood CHS, Wilson WJ, Malicka AN (2013). The effect of rTMS on auditory processing in adults with chronic, bilateral tinnitus: a placebo-controlled pilot study. Brain Stimul.

[36] Godbehere J, Sandhu J, Evans A (2019). Treatment of tinnitus using Theta burst based repetitive Transcranial magnetic stimulation-a single blinded randomized control trial. Otol Neurotol.

[37] Mennemeier M, Chelette KC, Allen S (2011). Variable changes in PET activity before and after rTMS treatment for tinnitus. Laryngoscope.

[38] Lee HY, Yoo SD, Ryu EW (2013). Short term effects of repetitive transcranial magnetic stimulation in patients with catastrophic intractable tinnitus: preliminary report. Clin Exp Otorhinolaryngol.

[39] Lorenz I, Müller N, Schlee W (2010). Short-term effects of single repetitive TMS sessions on auditory evoked activity in patients with chronic tinnitus. J Neurophysiol.

[40] Vanneste S, Ridder DD (2012). The involvement of the left ventrolateral prefrontal cortex in tinnitus: a TMS study. Exp Brain Res.

[41] Plewnia C, Vonthein R, Wasserka B (2012). Treatment of chronic tinnitus with θ burst stimulation: a randomized controlled trial. Neurology.

[42] Eichhammer P, Langguth B, Marienhagen J (2003). Neuronavigated repetitive transcranial magnetic stimulation in patients with tinnitus: a short case series. Biol Psychiatry.

[43] Bauer CA, Berry JL, Brozoski TJ (2017). The effect of tinnitus retraining therapy on chronic tinnitus: a controlled trial. Laryngoscope Investig Otolaryngol.

